# Fundamental Medical Mycology

**DOI:** 10.3201/eid1808.120521

**Published:** 2012-08

**Authors:** Thomas G. Mitchell

**Affiliations:** Duke University Medical Center, Durham, North Carolina, USA

**Keywords:** medical mycology, pathogenic fungi, antifungal drugs, fungi

In Fundamental Medical Mycology, Errol Reiss, Jean Shadomy, and Marshall Lyon have produced a valuable new text. Drs Reiss and Shadomy are medical mycologists who have extensive research and educational experience with the increasing spectrum of pathogenic fungi, including diagnosis of infections they cause and host defenses. Dr Lyon is an infectious diseases physician with clinical expertise in the epidemiology and management of opportunistic mycoses. This complementary team has produced a highly readable and comprehensive book, which they intend to be a text for medical and graduate students, a resource for microbiology technologists, and a reference for physicians and researchers.

The book has been carefully organized, and the extensive table of contents enables readers to quickly identify specific areas of interest. The first 3 chapters contain basic information describing fungi, diagnostic methods, and antifungal chemotherapy. The succeeding 19 chapters review specific mycoses, using a similar format that addresses the following topics: etiology, clinical manifestations, ecology of the fungi, epidemiology of the infections, pathogenicity, animal infections, treatment, and laboratory diagnosis. Each chapter provides instructive case histories and ends with references and review questions. The book also includes a helpful glossary.

A book of this nature can be judged by its completeness, accuracy and timeliness of coverage, clarity of the writing, and ease with which information can be accessed. By all of these criteria, Fundamental Medical Mycology merits high marks. The book does a superlative job in addressing recent advances in medical mycology, which include identifying emerging pathogens, new antifungal drugs and strategies for their use; progress in molecular diagnostics; and up-to-date knowledge about host defenses against fungi, especially opportunistic pathogens. For a 1-volume text, this book provides excellent coverage of several critical areas: detailed methods of identifying fungi; descriptions of common and rare mycoses; the nuances of interpreting serologic tests; and ongoing progress in detecting diagnostic fungal antigens, nucleic acids, and signature proteins in clinical specimens.

In addition, the authors provide superb, concise descriptions of the strain diversity of the major pathogenic species and the clinical and epidemiologic relevance of certain phylogenetic clades. Currently unresolved or controversial topics are clearly explained, such as fungal sinusitis and the etiology of *Malassezia* spp. infections. When information is available, each chapter summarizes the mechanisms of pathogenicity and confirmed virulence factors. The authors discuss advances in understanding the innate and adaptive immune responses to fungi at the tissue, cellular, and molecular levels (e.g., the role of Th17 immune responses in candidiasis). Another asset is the frequent but unobtrusive inclusion of key citations to assist anyone seeking additional information. The illustrations include diagrams, clinical photographs, and photomicrographs from a variety of sources as well as original figures. Rather than attempt pictorial consistency throughout, the authors have gleaned images for their relevance to the text.

This book will serve medical and graduate students who will value the book’s succinct, lucid coverage of key fungal infections, as well as instructive case vignettes and review questions. Clinical fellows and physicians will appreciate the readable summaries of specific mycoses, diagnostic procedures, common symptoms, and appropriate antifungal drugs. Biomedical scientists and educators in related fields will use this text as a resource for a quick review of specific topics.

